# Short-term sedation of mechanically ventilated ICU patients with propofol, benzodiazepines, or dexmedetomidine: systematic review and meta-analysis on awakening and recovery times

**DOI:** 10.1186/cc14566

**Published:** 2015-03-16

**Authors:** A Feuersenger, L Pradelli, A Aliano, JF Baron, M Westphal

**Affiliations:** 1 Fresenius Kabi Deutschland GmbH, Bad Homburg, Germany; 2 AdRes, Torino, Italy; 3 Fresenius Kabi ELAMA, Paris, France

## Introduction

Sedation in the ICU is crucial in reduction of patients' discomfort, in particular in patients undergoing mechanical ventilation to help tolerate intubation and reduce pain and anxiety. Propofol (Pr) is a widely used option, but other viable alternatives for short-term sedation (STS; that is, <24 hours) include benzodiazepines (BDZ) and dexmedetomidine (Dx). We aimed at pooling all available evidence on the comparative effects of Pr in terms of awakening and recovery times after STS in mechanically ventilated ICU patients.

## Methods

We planned a systematic literature review searching Medline and Scopus and performed a meta-analysis on direct comparisons reporting on weaning time (Tw), duration of mechanical ventilation (Tmv), time to extubation (Tex) and length of stay in the ICU (Ticu). The primary analysis considered only data from RCTs, while in a secondary analysis observational studies were also included.

## Results

The literature search identified 15 relevant RCTs, of which 11 versus BDZ, and a further five observational studies, of which one versus BDZ. When compared with BDZ, Pr associated with significantly reduced Tw (-1.6 hours, 95% CI: -2.5 to -0.8), Tmv (-2.0 hours, 95% CI: -3.7 to -0.2), and Ticu (-5.0 hours, 95% CI: -8.5 to -1.4); no statistically significant difference resulted when comparing Pr and Dx. When nonrandomized evidence was included, results did not change significantly.

## Conclusion

In conclusion, Pr is associated with shorter awakening and recovery times after STS than BDZ, while no difference could be shown when Pr was compared with Dx.

